# *Molecular Oncology* journal has moved to open access

**DOI:** 10.1002/1878-0261.12031

**Published:** 2017-02-01

**Authors:** Julio E. Celis,, Jose Moreira,

*Molecular Oncology* has become fully Open Access in 2017 joining the other FEBS journals on the FEBS Press platform and Wiley. From January 2017, all articles in *Molecular Oncology* are published under the Creative Commons Attribution License (CC BY), allowing readers to access the journal irrespective of speciality, host institution or location.

The journal will continue to highlight new discoveries, approaches, as well as technical developments, in basic, clinical and discovery‐driven translational cancer research. Priority is given to work that significantly advances our understanding of disease processes leading to human tumour development and establishes novel concepts of clear clinical significance in diagnosis, prognosis and prevention strategies. We publish Research articles, Science Policy reports, Reviews (by invitation only) and Thematic issues (by invitation only). Science Policy reports, Reviews and Thematic issues are free of charge. For more information, see http://febs.onlinelibrary.wiley.com/hub/journal/10.1002/ (ISSN)1878-0261/index.html.

To keep abreast of new developments, we have recently appointed Ulrik Ringborg (Cancer Center Karolinska, Stockholm) as Senior Associated Editor and four new members of the Editorial Board: Christopher Lord (The Institute of Cancer Research, London), Daniel Peeper (The Netherlands Cancer Institute, Amsterdam), Stefano Piccolo (University of Padua School of Medicine, Padua) and Joan Seoane (Vall d'Hebron Institute of Oncology, Barcelona). Their expertise, together with that of the rest of the Editorial Board (http://febs.onlinelibrary.wiley.com/hub/journal/10.1002/(ISSN)1878-0261/editorial-board.html), will help shape the future development of the journal.

In the new Open Access format, we expect the editorial team to continue to apply the same rigorous standards of peer review and acceptance criteria, ensuring that the journal remains the high‐quality publication we have come to value greatly.

We very much hope that you share our excitement for these new developments for *Molecular Oncology* and if you have any questions, please do not hesitate to contact us.

Finally, we would like to thank Elsevier for the continuous support and encouragement we received during the past ten years. In particular, we would like to thank Adrian Klinkenberg, Carl Schwarz, Lucia Muñoz Franco and Olaf Meesters with whom we worked closely together for various periods of time during the development of the journal. Also, our thanks extend to Mary Purton from FEBS Press as well as members of the Wiley team for the much‐appreciated help and advice received during the transition period.

